# Dysbiosis of the enteric DNA virome correlates with the development of cachexia in a murine Lewis lung carcinoma (LLC) model

**DOI:** 10.1007/s00705-026-06522-7

**Published:** 2026-02-22

**Authors:** David Aciole Barbosa, Yara N.L.F. de Maria, Fabiano B. Menegidio, Regina Costa de Oliveira, Daniela L. Jabes, Luiz R. Nunes

**Affiliations:** 1 EasyOmics Biotechnology, Mogi das Cruzes, Brazil; 2 Núcleo Integrado de Biotecnologia, Universidade de Mogi das Cruzes, Mogi das Cruzes, Brazil; 3 Núcleo de Pesquisas Tecnológicas, Universidade de Mogi das Cruzes, Mogi das Cruzes, Brazil; 4 Centro de Ciências Naturais e Humanas, Universidade Federal do ABC, Santo André, Brazil

**Keywords:** Cachexia, Gut inflammation, Virome, Metagenome, NGS, Giant viruses, Phycodnaviridae, Bacteriophages, Microviridae, Inoviridae

## Abstract

Cachexia, a multifaceted wasting syndrome, profoundly impacts quality of life and survival rates in cancer patients. Gut inflammation is identified as a key player among the contributing factors for its development. Consequently, numerous studies have sought to characterize changes in gut microbiota of cachectic individuals, given the well-established roles of the gut microbiota in controlling and/or triggering both local and systemic inflammation in their hosts. Most of these investigations have applied mouse models of tumor-induced cachexia to show correlations between alterations in bacterial and fungal abundance in the digestive tract and the onset of cancer cachexia (CC). However, the role of viral dysbiosis in CC development remains unexplored. The present study aims to address this gap by characterizing the gut virome during the progression of murine cancer cachexia. Although our approach was limited to DNA viruses, our findings reveal that cachectic animals with Lewis lung carcinoma exhibited a subtle yet statistically significant modulation in composition (R^2^ = 0.17622; *p* = 0.05). A linear discriminant analysis effect size (LEfSe) analysis revealed that the dysbiosis observed in the gut virome of CC animals was mostly characterized by a significant enrichment in giant viruses of the family *Phycodnaviridae* (LDA score, 4.2582; *p*-value, 0.004; pwr_app_, 0.9984) and significantly decreased populations of bacteriophages of the families *Microviridae* (LDA score, 4.3458; *p*-value, 0.0127; pwr_app_, 0.9065) and *Inoviridae* (LDA score, 3.3028; *p*-value, 0.0017; pwr_app_, 0.9992). This cachexia-associated viral dysbiosis shares similarities with virome alterations documented in other conditions linked to gut inflammation, including, ulcerative colitis, Crohn’s disease, and *Clostridioides difficile* infection. These new insights suggest the potential contributions of viral communities to the pathophysiology of CC and other inflammation-driven diseases.

**Supplementary Information:**

The online version contains supplementary material available at 10.1007/s00705-026-06522-7.

## Introduction

Cachexia, a multifactorial metabolic syndrome, associates generalized chronic inflammation with severe weight loss, due to the continuous consumption of muscle and adipose tissues in affected individuals. It is frequently observed in association with chronic kidney/heart diseases, as well as several types of cancer (especially in advanced stages), contributing to reduced quality of life and life expectancy [1–4]. Efforts to treat cachexia are often unsuccessful due to the patient's debilitated state and the syndrome's multiple and complex drivers. Among these, persistent gut inflammation – apparently linked with intestinal dysbiosis – is a significant contributing factor [5, 6].

Most findings linking gut dysbiosis and cachexia development have been supported by experiments employing mouse models in which cachexia is induced by inoculation with tumor cells. Such studies have consistently demonstrated strong correlations between alterations in the relative abundance of various bacteria [6–9] and fungi [10] in the digestive tract and the onset of cancer-induced cachexia (CC).

However, studies regarding the influence of different components of the gut microbiota on the development of CC have not examined differences in composition regarding viral populations. Nonetheless, recent research has begun to shed light on the influence of virome alterations on various diseases [11]. The virome includes viruses that are capable of infecting bacteria (bacteriophages), archaea (archaeal viruses, or archeaophages), and eukaryotes (eukaryotic viruses). Thus, its components can interact not only with the host itself but also with other members of the microbiome, playing a pivotal role in maintaining homeostasis [12]. Not surprisingly, alterations in the virome have been identified as contributing factors in conditions involving the gastrointestinal tract (inflammatory bowel disease and colorectal cancer) [13, 14], respiratory tract (asthma and chronic obstructive pulmonary disease [15, 16]), and the nervous system (multiple sclerosis) [17], among others [18].

Thus, we hypothesized that the development of CC could also be associated with dysbiosis of the gut virome. Given the profound gut inflammation and barrier disruption observed in CC, we postulated that such a dysbiosis could contribute to pathogenesis through several non-exclusive pathways, including a shift in the bacteriophage community that disrupts gut microbiome stability and an enrichment of free viral particles in the gut, which could potentially engage the host immune system and trigger both local and systemic inflammatory responses. To test this hypothesis, we conducted a metagenomic analysis of the fecal virome in LLC-bearing mice – a model that has already been employed to verify both bacterial [9] and fungal [10] CC-related dysbioses. Our results revealed a distinct viral dysbiosis in cachectic animals, which was characterized by a shift in viral community structure, including depletion of certain core bacteriophage families (*Microviridae* and *Inoviridae*) and an expanded representation of eukaryotic giant viruses (*Phycodnaviridae*). These findings provide the first evidence of specific virome alterations in this CC model and suggest a potential role for the gut virome in the pathophysiology of cancer cachexia.

## Material and methods

### CC induction in C57BL/6 mice

Six- to eight-week-old male C57BL/6 mice (Jackson Laboratory), weighing ~ 17–24 g (mean: 22.7 g) were used in this study. The exclusive use of male mice is standard practice in studies involving tumor-induced cachexia, improving experimental consistency by minimizing hormonal variability among subjects. Female mice exhibit cyclic fluctuations in estrogen and progesterone levels that significantly affect their overall metabolism, immune responses, and muscle/adipose tissue dynamics, which can confound the interpretation of cachexia-related outcomes. Male mice, on the other hand, tend to develop more-pronounced and homogeneous cachectic responses to tumor inoculation, facilitating standardization and comparison with the existing literature on CC-related dysbiosis [6–10, 19].

All animal procedures were approved by the University of Mogi das Cruzes Animal Ethics Committee (approval no. 012/2017). Four- to six-week-old male C57BL/6 mice were housed in a controlled, rodent-only environment (24 ± 1 °C, 12-hour light/dark cycle) with *ad libitum* access to irradiated food (Nuvilab CR1-irradiated; Nuvital) and sterile, autoclaved water. To prevent aggression, male mice were housed in small groups of four animals per cage. To overcome the high degree of inherent individual variability in their gut microbiota (a major confounding factor in murine studies), a previously established cage rotation protocol was implemented [9, 10, 20]. Cage rotation is a simple and widely employed strategy to normalize baseline microbiota variation across entire cohorts [21–23]. Briefly, it involves systematically rotating mice between cages every two days during a two-week period. This ensured that all animals were exposed to the bedding, feces, and environmental droppings of all other mice in the cohort. The natural coprophagic behavior of mice facilitates direct microbial exchange, a known driver of microbiota homogenization [24], thereby standardizing the microbial baseline and reducing pre-experimental variation between individuals.

After microbiota synchronization, 10 mice were randomly selected as a control (CT) group and injected subcutaneously with 200 µL of saline solution in the right flank. The remaining 20 mice were injected similarly with 3.5 × 10^5^ LLC tumor cells in 200 µL of Dulbecco’s modified Eagle medium. Animals were then housed in pairs in sterile cages under the same environmental conditions and observed for 28 days, when CC development was assessed. Among the LLC-injected animals, we selected mice showing five pre-defined hallmark symptoms of cachexia: (i) cachexia index (CI) > 5 [4, 25], (ii) gastrocnemius muscle mass loss, (iii) increased expression of muscle atrophy and inflammation markers (atrogin and IL6R), and (v) histometric alterations in adipose tissue (see reference 10 for details). Stool samples from animals in the cancer cachexia [CC] group were collected on day 28 and used for subsequent microbiome analysis. Samples from a similar group of mice from the CT group (also collected on day 28) were randomly selected to serve as the saline control (SC) group.

Lewis lung carcinoma cells, obtained from ATCC (catalog no. CRL-1642), are routinely maintained in our facilities and monitored monthly for mycoplasma contamination, using a Venor GeM Mycoplasma Detection Kit (Minerva Biolabs), following the manufacturer’s protocol. Only cultures confirmed to be mycoplasma-free were used for subsequent experiments.

### Virus isolation, DNA/RNA extraction, amplification, and NGS sequencing

Virome libraries were prepared according to the Netovir protocol [26]. Briefly, stool samples were homogenized in 300 µL of 1% PBS in a Minilys Personal Homogenizer at 3000 rpm for 1 minute and then centrifuged at 17,000 *g* for 3 minutes. The supernatant was filtered by two passages through 0.45-µm and 0.22-µm PVD filters. The eluate was treated with benzonase (Milipore/Sigma, catalog no. E1014-5KU) and micrococcal nuclease (NEB, catalog no. M0247S) to degrade genomic material not protected by viral capsids. DNA/RNA was then extracted and purified using a QIAmp Viral RNA Mini Kit (QIAGEN, catalog no. 52904), following the manufacturer's protocol, without RNA carrier. Nucleic acids were quantified using a Quantus fluorimeter, using QuantiFluor dsDNA and RNA System kits (Promega, catalog nos. E2671 and E3310).

Next, reverse transcription and amplification of cDNA were performed using a WTA2 kit (Sigma, catalog no. WTA2-10RXN), and the product was purified using a QIAquick PCR Purification Kit (QIAGEN, catalog no. 28104). Libraries were constructed using an Illumina Nextera XT Kit (Illumina catalog no. FC-131–1024). Samples (3 ng of cDNA/DNA) were tagged at 55 °C for 4 minutes, indexed, and amplified for 15 cycles in a thermocycler (MJ Research PTC-100). Library quality and fragment sizes were evaluated using an Agilent Bioanalyzer 2100 with a Kapa Library Quantification Kit (Kapa Biosystems catalog no. KR0405) and pooled for sequencing on an Illumina NextSeq DNA sequencer. Raw sequences were deposited in the OSF database (https://osf.io/at3xz).

### NGS sequence processing

Sequence read quality was evaluated using FastQC and MultiQC on Galaxy.eu [27]. Trimmomatic was used for quality filtering (Q ≥ 30; minimum length, 50 bases) [28]. Reads were mapped to the *Mus musculus* mm10 genome using BWA-MEM [29], and host sequences were removed using Samtools view and SamToFastq [30].

Metagenomes were assembled using MEGAHIT [31] (min-count = 2, k-min = 21, k-max = 99, and k-step = 20). CD-HIT-EST [32] was used to filter redundancy. From these representative contigs (“repcons”), sequences longer than 1 kb were analyzed using Transdecoder to predict ORFs [33]. These ORFs were then annotated through a protein similarity search against VOGdb (v.6.0), using Diamond [34]. Finally, an ORF density filter was used to exclude repcons containing ≤ 1 ORF/4 kb, resulting in a final annotated dataset of 8,840 ORFs across 6,664 repcons.

Taxonomic classification of repcons was assigned at the family level using TaxonKit, with a voting scheme for reliability [35]. Further metadata (genetic material type and host) were retrieved from ICTV’s Virus Metadata Resource (VMR) v.5 and Master Species List (MSL) v1010.v1 tables. The complete workflow is shown in ESM_1.

### Virome analysis in CC and SC animals

To assess the abundance of viruses sampled in the intestines of SC and CC animals, reads free of host material were mapped to repcons, using Bowtie2 [36], and the raw number of mapped reads was obtained using the Samtools idxstats tool [30]. Raw counts were normalized in an R [37] environment with the aid of a customized script (based on the script provided in reference 38, using the RPKM transformation (reads per kilobase of transcript, per million mapped reads) (scripts found in ESM_3). Viral abundance was quantified using reads per kilobase per million mapped reads (RPKM). This metric was selected for its applicability to viral metagenomics, where reference-based methods face specific challenges. Tools such as Kraken 2 [39] provide raw counts biased by genome length, while more-advanced methods such as Bracken [40] and the TPM metric [41] output relative abundances. Thus, both are constrained to a constant sum, which introduces compositional bias and makes the apparent abundance of any virus dependent on the abundance of all others in the sample [42]. Furthermore, these methods rely on complete reference genomes, and the pervasive incompleteness and fragmentation of viral reference databases lead to significant inaccuracies in abundance estimation for novel or partially represented viruses [38]. In contrast, RPKM provides a measure of normalized coverage for our assembled contigs without a constant-sum constraint [43], ensuring transparent quantification. This approach is in accord with established practices in exploratory virome research, as demonstrated in a substantial number of seminal articles published over the past decade [44–51].

Taxonomic classification was combined with the RPKM-transformed counts to create a virus-OTU-like table (vOTU-like table) in tabular format (.txt). This file was processed locally, in a Unix system, with QIIME v1.9.1 [52], using the convert function of the biom-format package [53] to obtain a.biom format and create a vOTU-like table with 6,664 vOTUs. For alpha and beta diversity analysis, the.biom files were subjected to minimum count filtering with the filter_otus_from_otu_table script, maintaining only 5,699 vOTUs, presenting at least 0.001% abundance. This vOTU-like table was loaded into the MicrobiomeAnalyst platform [54], using the following parameters: "minimum count = 0"; "prevalence in samples (%) = 10"; "percentage to remove (%) = 0"; do not rarefy my data"; "do not scale my data"; and "do not transform my data". The virus community profiles of the SC and CC groups were compared through alpha-diversity analysis to assess variations in community richness and diversity [55] at the family level, using the T-test (see ESM_4 for normality tests; ESM_5 for metadata map) with *p* ≤ 0.05 as a threshold. Beta diversity was explored using the Bray-Curtis distance [56] in a principal coordinates analysis (PCoA) [57], verified using a permutational multivariate analysis of variance (PERMANOVA) test, combined with permutational multivariate analysis of dispersion (PERMDISP) to show that the difference was not due only to sample dispersion [58, 59]. Results from beta-diversity analysis also employed *p* ≤ 0.05 as threshold. Finally, the filtered vOTU-like table was processed with the compute_core_microbiome script to retain only OTUs present in at least 70% of the samples in each experimental group, and summarize_taxa script, to provide a vOTU-like table in tabular format. This file was then transformed using a custom script created to convert it to the input format required for the linear discriminant analysis effect size (LEfSe) program [60]. The file was then loaded into the Galaxy Huttenhower lab environment (http://huttenhower.sph.harvard.edu/galaxy/) of LEfSe, which identified significantly discriminative taxa between the SC and CC groups, using a modular value for logLDA ≥ 2 and *p* ≤ 0.05 as threshold criteria. Finally, we calculated the approximate statistical power (pwr_app_) for each differentially abundant viral family, using an abundance of core70 vOTU-like table and Cliff’s delta (δd), following established protocols for metagenomic data [61, 62], as suggested previously [63]. All scripts and data (raw and processed) employed in the analyses described in this article are available through OSF.

### Ethics statement

All animal work was approved by the University of Mogi das Cruzes Animal Ethics Committee (approval number 012/2017).

## Results

### General virome characterization

C57BL/6 mice underwent LLC tumor cell transplantation and were monitored for 28 days under controlled conditions. At the end of this period, eight animals exhibiting characteristic signs of advanced cachexia were selected. These animals were designated as the cancer cachexia (CC) group. For comparison, we randomly selected a matching group of healthy saline-injected mice to serve as the saline control (SC) group for subsequent analysis.

Viral particles were extracted from fecal samples from both CC and SC animals, and their genetic material was purified and quantified. At the end of this procedure, we were able to obtain detectable amounts of DNA/RNA material for only seven CC animals. Thus, libraries were prepared for 14 animals (seven from the CC group and seven from the SC group), which were subjected to next-generation sequencing (NGS) in an Illumina NextSeq DNA sequencer, yielding ~ 68 million reads (ESM_2).

**Fig. 1:**
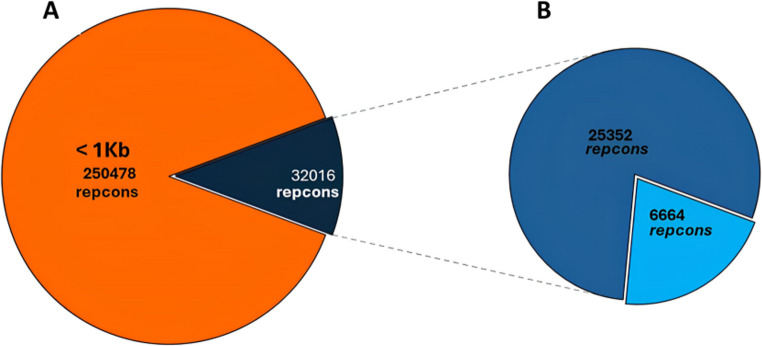
Assembly and annotation of virome in mouse gut. **A** A total of 67,596,881 NGS reads obtained from mice gut virus material were assembled into 250,511 representative contigs (repcons). **B** Repcons ≥ 1 kb (32,016) were analyzed using Transdecoder to find ORFs, which were then compared to the VOGdb database, identifying virus-specific sequences, which were then used to assign taxonomic identification to 6,664 repcons at the family level, with the aid of TaxonKit

Viral metagenomic contigs were assembled from these reads using MEGAHIT [31] and CD-HIT-EST [32], resulting in 250,511 representative contigs (repcons) (Fig. 1A). Repcons longer than 1 kb (32,016) were analyzed using Transdecoder, identifying 131,181 ORFs (average of ~ 4 ORFs per repcon). ORFs were then annotated by protein similarity against the VOGdb database (v.6.0), using Diamond, resulting in 6,664 repcons, containing 8,840 annotated ORFs (Fig. 1B).

Thus, our final reference dataset consisted of ~ 21% of the originally assembled repcons ≥ 1 kb (6,664/32,016), confirming previous studies that suggested the existence of a large number of unique DNA sequences in viral populations (the so-called “viral dark matter”) [64].

Annotated ORFs were used to attribute taxonomic classification to the reference repcons, using TaxonKit [35]. However, given the reduced number of annotated ORFs in the final set of repcons (average of ~ 1.3 annotated ORFs per repcon) and the significant amount of dark matter in their sequences, we opted to attribute taxonomic classification at the family level only.

**Fig. 2:**
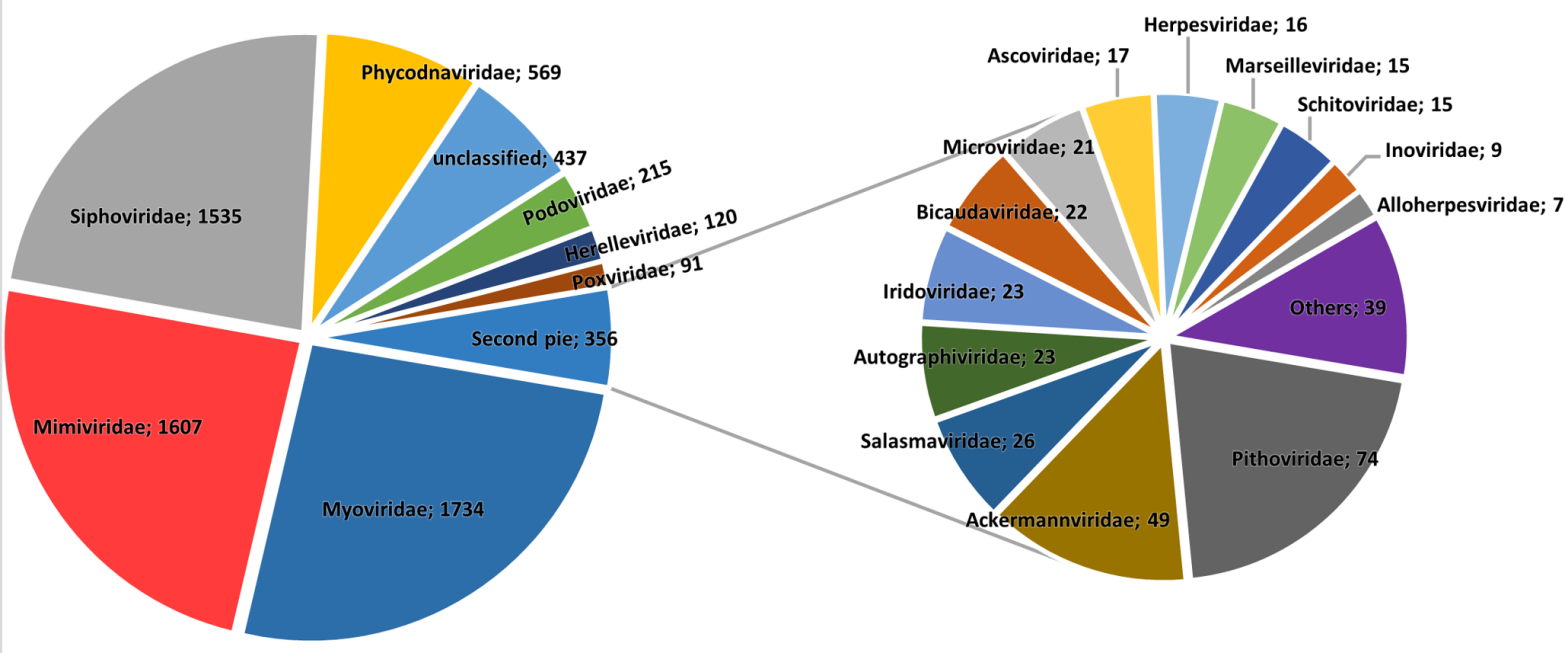
General gut virome composition of the mice used in the study. The figure shows the distribution of 6,664 viral elements (repcons) detected in the intestinal virome of both SC and CC mice across the main viral families, highlighting the absolute number of repcons associated with each of them

Most of the viral repcons found in the stool samples of the mice corresponded to members of the former bacteriophage families "*Myoviridae*" (~ 26%), "*Siphoviridae*" (~ 23%), and "*Podoviridae*" (~ 3.2%) and the current family *Herelleviridae* (~ 1.8%), with smaller proportions corresponding to the families *Ackermannviridae* (~ 0.73%), *Salasmaviridae* (~ 0.4%), *Microviridae* (~ 0.34%), *Schitoviridae* (~ 0.22%), and *Inoviridae* (~ 0.13%), and the former family "*Autographiviridae*" (~ 0.34%), among others. We also found eukaryotic viruses of the families *Mimiviridae* (~ 24.1%), *Phycodnaviridae* (~ 8.5%), *Poxviridae* (~ 1.4%), *Pithoviridae* (~ 1.1%), *Iridoviridae* (~ 0.35%), *Ascoviridae* (~ 0.25%), *Marseilleviridae* (~ 0.22%), *Alloherpesviridae* (~ 0.1%), and the former family "*Herpesviridae*" (~ 0.24%). Interestingly, a large proportion of repcons (~ 34.5% of the total virome) is associated with giant viruses. Finally, our dataset also contained a few sequences from viruses capable of infecting archaea, including representatives of the families *Bicaudaviridae* (~ 0.33%), *Lipothrixviridae*, *Rudiviridae* (only two repcons each), *Ampullaviridae*, *Sphaerolipoviridae*, and *Spiraviridae* (only one repcon each) (Fig. 2).

### Comparative analysis of the viromes of SC and CC mice

Sequences from repcons were used as references to align the NGS reads obtained from SC and CC, providing estimates of their relative abundance. This information, along with the taxonomic assignment for each annotated repcon, was consolidated in a virus-OTU-like table (vOTU-like table), which was then filtered to remove elements present at ≤ 0.001% abundance (ESM_6).

**Fig. 3:**
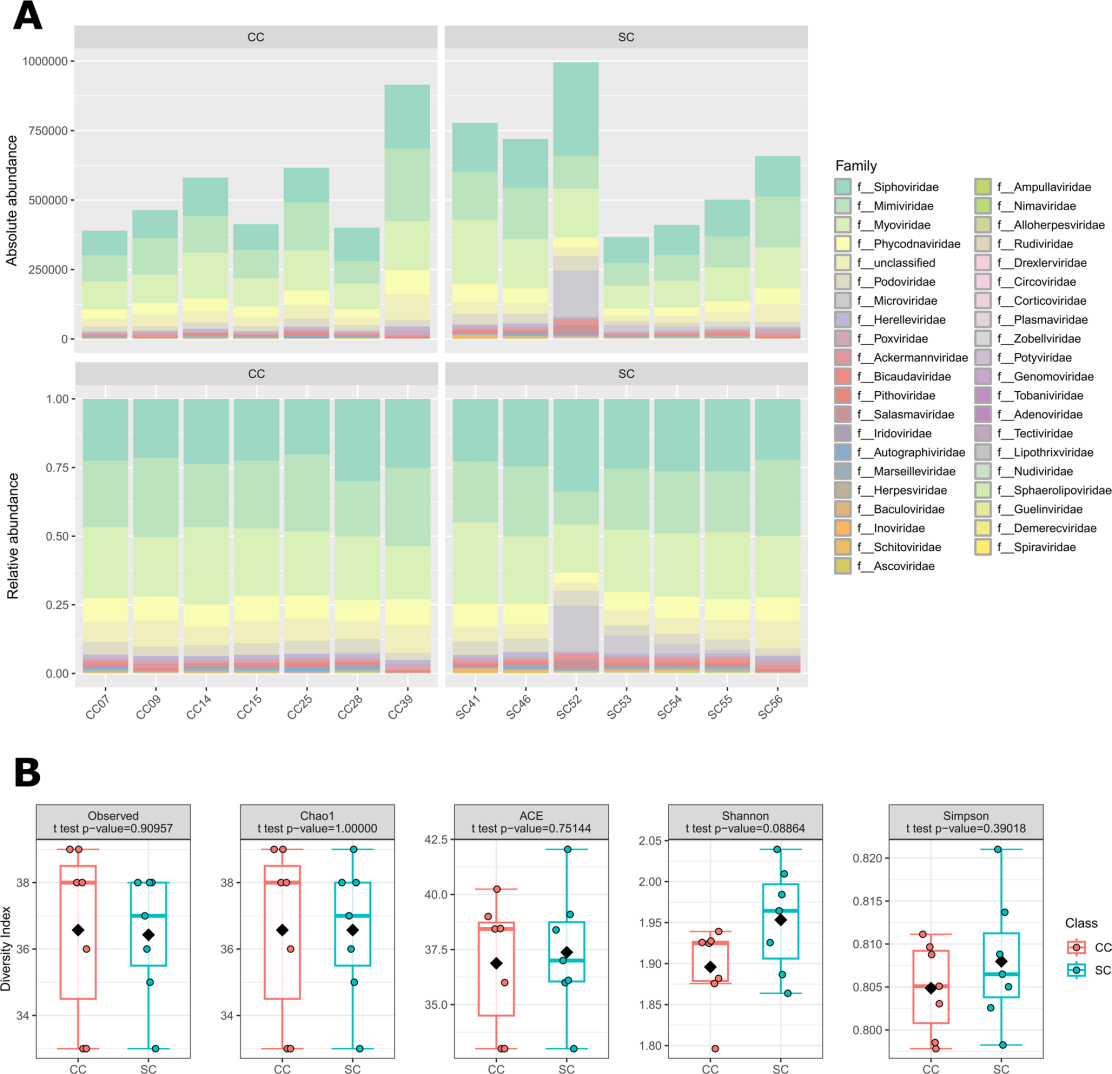
Alpha diversity comparing mice of the SC (blue) and CC (red) groups. **A** Contribution of different viral families to the virome composition in each mouse. **B** Absolute observed vOTU (repcon) counts (n = 7 animals per group; T-test: *p* = 0.90957). **C** Chao 1 index (*n* = 7 animals per group; T-test: *p* = 1.00000). **D** ACE index (*n* = 7 animals per group; T-test: *p* = 0.75144). **E** Shannon index (n = 7 animals per group; T-test: *p* = 0.08864). **F** Simpson index (*n* = 7 animals per group; T-test: *p* = 0.39018). Statistically significant differences identified by T-test, using *p* ≤ 0.05 as a threshold

A preliminary comparison of gut virome composition between SC and CC animals showed that both are dominated by myophages, siphophages, and members of the families *Mimiviridae* and *Phycodnaviridae*), along with members of several underrepresented families, which may contribute to differentiating the two microbiomes in richness, diversity, and composition (Fig. 3A). Initially, we conducted alpha-diversity analysis using the absolute number of repcons observed in each group (Fig. 3B). We also calculated Chao 1 and ACE indexes, which indicate the presence of rare taxa in alpha-diversity analysis [55, 59] (Fig. 3C and D). As shown in Figs. 3B to D, none of these indicators show statistically significant (*p* > 0.05) variation in richness. Furthermore, no statistically significant difference in viral diversity was observed between the SC and CC viromes based on their Shannon and Simpson indexes [55, 59] (Fig. 3E and F).

**Fig. 4:**
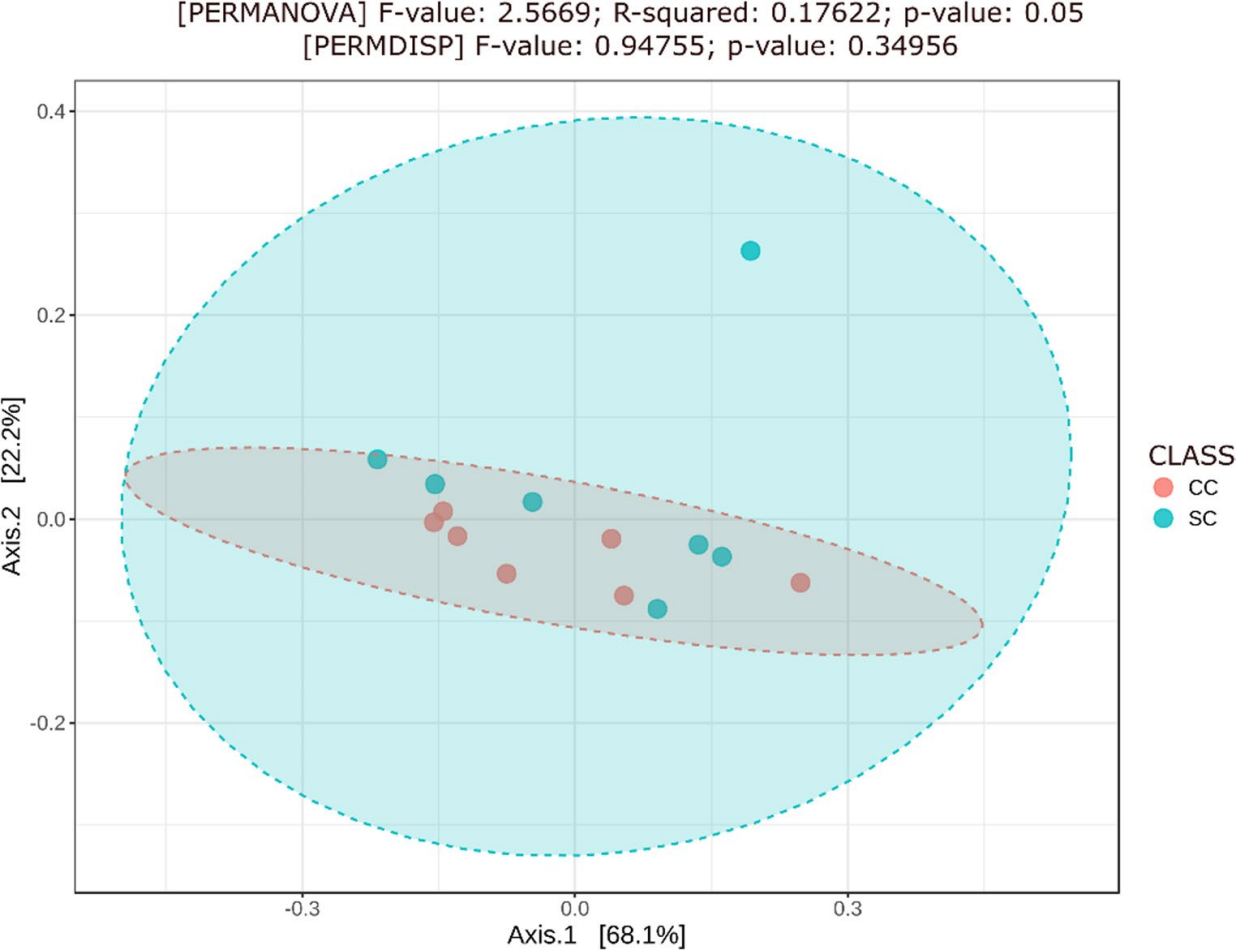
Beta diversity analysis comparing mice from the SC (in blue) and CC (in red) groups. The difference in composition between the viromes of the two groups was estimated and compared using a principal coordinates analysis (PCoA) algorithm, followed by statistical validation by PERMANOVA, using a *p*-value ≤ 0.05 as the significance threshold. A PERMDISP analysis, also using a *p*-value ≤ 0.05 as the significance limit, demonstrated that the difference in composition detected between the viromes of the two groups is not an artifact derived from differences in sample dispersion from the two groups.

Next, to confirm that the SC and CC viromes differ in composition, a beta diversity analysis was performed, with the aid of a Bray–Curtis PCoA, followed by statistical validation by PERMANOVA (Fig. 4). This analysis indicated a modest differentiation in composition between the gut viromes of SC and CC mice, since the *R*^2^ value of 0.17622 indicates that the group effect explains around 18% of the variation in the dataset (Fig. 4). Nonetheless, this compositional variation is statistically significant (*p* = 0.05) and is not due to differences in sample dispersion, as verified by a PERMDISP analysis (*p* = 0.34956), supporting the hypothesis that there is no difference in sample dispersion between the groups (Fig. 4) [58, 59].

Given the borderline significance of our beta-diversity analysis (*p* = 0.05), the viromes of SC and CC were further compared using LEfSe, a widely used tool for identification of differentially represented elements from microbiome data [60]. This was conducted with a “core 70%-v-OTU-Like table”, which was further filtered to retain only elements present in at least 5 (~ 70%) of the animals in each group (ESM_7; see Materials and methods for details). The use of core OTU tables, i.e., retaining only those OTUs present in a defined percentage of subjects within each experimental group, is a well-recognized method to improve reproducibility, reduce noise, and focus on taxa that are both prevalent and biologically meaningful [65]. Multiple studies have shown that filtering out rare or low-prevalence OTUs improves detection reliability across replicates, stabilizes beta-diversity estimates, and reduces technical variability [66–71]. We therefore used core-70 OTU tables following the recommendation of Risely et al. [65], who demonstrated that community ecological patterns remain stable up to approximately 70% prevalence thresholds, beyond which comparisons may become distorted and less representative of the full community structure. This value is in accord with earlier microbiome-based investigations [9, 10, 66, 72–74].

**Fig. 5:**
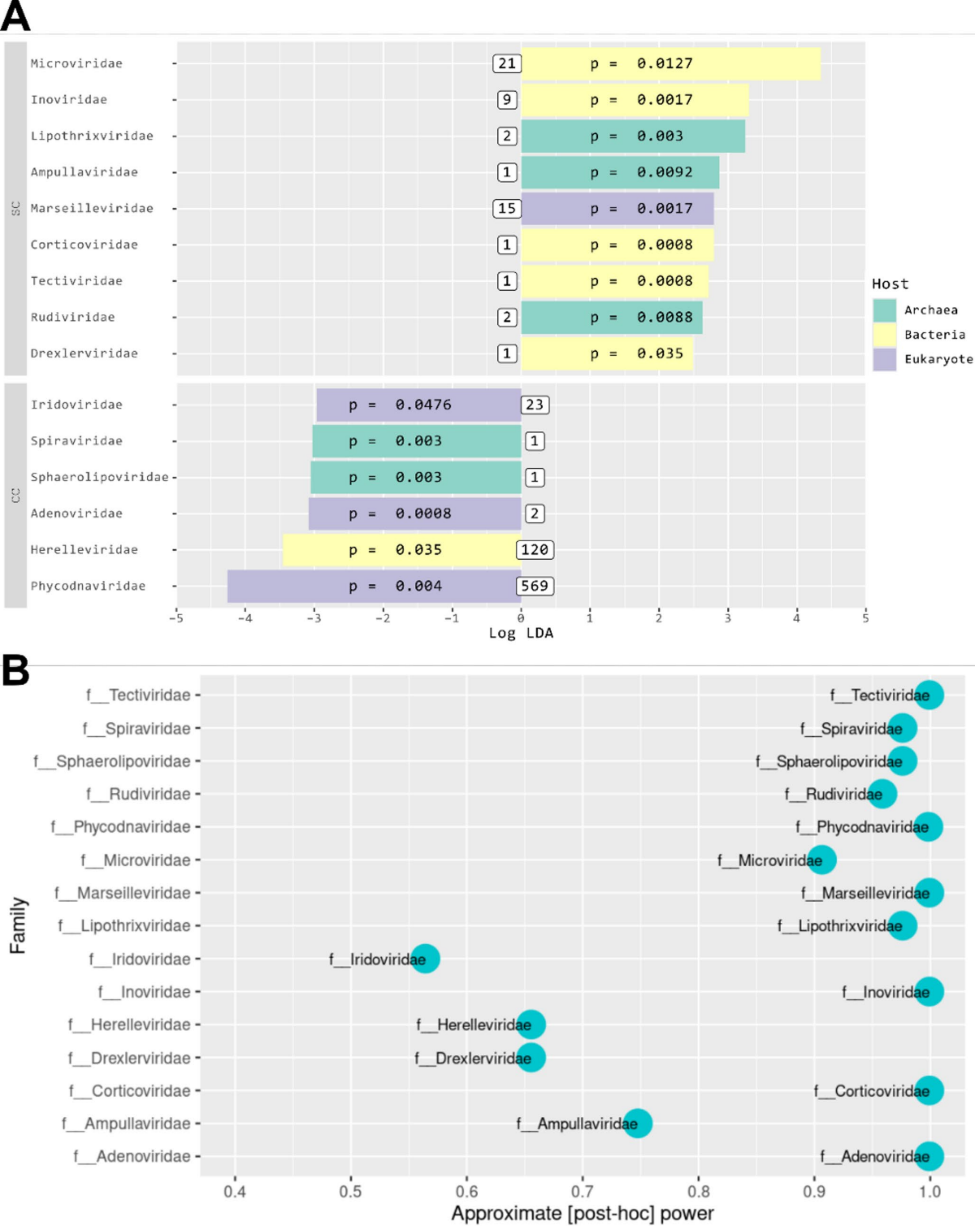
**A** Identification of families differentially represented between the microbiotas of SC (blue) and CC (red) animals through LEfSe analysis. This analysis used a *p*-value ≤ 0.05 and a modular threshold of LDA score ≥ 2 (−2 ≤ LDA score ≥ 2) as stringency criteria for the identification of families differentially represented in the CC and SC groups. The type of host and number of repcons present in each differentially represented family are shown next to each bar, totaling 767 elements (11.5% of the total repcons originally detected in the mouse virome) (Fig. 2). **B** Approximate post-hoc power based on the abundance in the core70 vOTU-like table

LEfSe analysis revealed distinct viral family representations between the stool viromes of the two mouse groups. The virome of the control group (SC) was characterized by a higher proportion of bacteriophages, primarily members of the families *Microviridae* (small, single-stranded DNA phages) and *Inoviridae* (filamentous phages), as well as a few archaeal viruses (archaeophages). For eukaryotic viruses, only the giant-virus family *Marseilleviridae* was overrepresented in SC animals. In contrast, the virome of the cachectic group (CC) showed a marked overrepresentation of eukaryote-infecting giant viruses, particularly of the families *Phycodnaviridae* and *Iridoviridae*. Notably, the only bacteriophage family overrepresented in CC animals was *Herelleviridae* – a group specialized in infecting *Firmicutes* bacteria [75] (Fig. 5A).

Finally, given the modest sample size of this study (seven animals per group), we assessed the approximate statistical power (pwr_app_) for each differentially abundant viral family. This was done using the abundance of core70 vOTU-like table and Cliff’s delta (δd), following established protocols for metagenomic data [61, 62], as suggested by Ferdous et al. [63]. This analysis confirmed enough power (pwr_app_ > 0.9) for 11 of the 15 differentially abundant taxa (Fig. 5B).

**Fig. 6:**
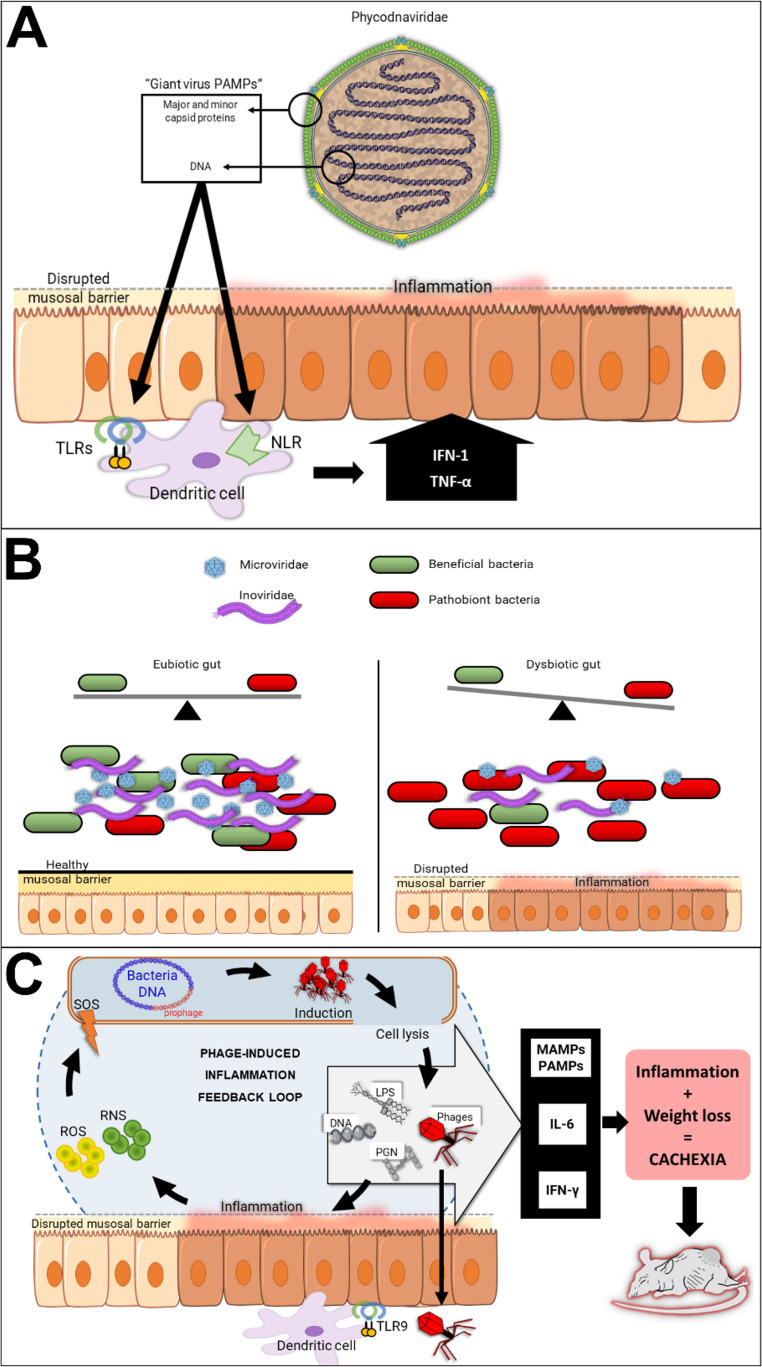
Possible involvements of viruses in cachexia syndrome. **A** Capsid proteins and DNA can function as “giant virus PAMPs” that induce IFN-1/TNF-α via TLRs and NLRs. **B** Reduced *Microviridae* and *Inoviridae* populations may drive unchecked proliferation of pathobionts or specific bacterial consortia that contribute to inflammation and metabolic dysfunction. **C** Phage-induced inflammation feedback loop. ROS and RNS enhance lysogeny, reactivating the lytic cycle via the SOS response. MAMPs and PAMPs such as lipopolysaccharides, DNA, and peptidoglycan, reinitiate the loop while phages interact with TLR9 resulting in pro-inflammatory cytokines. Progressive inflammation and weight loss are key points in cachexia.

Such alterations in virome composition might constitute an inflammation feedback loop, as shown in Fig. 6.

## Discussion

Cachexia is one of the most devastating clinical conditions that can occur in individuals affected by various types of cancer, as its high morbidity significantly reduces life quality and expectancy. Moreover, cachexia is a multifactorial syndrome that is challenging to combat, as it is associated with chronic inflammation, as well as various internal and external factors [76]. In this context, the correlation between cachexia development and intestinal dysbiosis, as observed in mice, has led researchers to hypothesize that manipulation of gut microbiota may provide a viable therapeutic target for preventing and/or mitigating CC development in cancer patients [77].

Unfortunately, that studies that have been conducted so far suggest significant variability in the nature of this dysbiosis, depending on the type of tumor used in experimental models, while focusing exclusively on the characterization of bacterial and fungal gut microbiotas [6]. In this context, the results presented here represent a pioneering effort, leading to the identification of 6,664 viral repcons in fecal material collected from CC animals, of which approximately 11.5% (767) exhibited significant populational changes in cachectic animals when compared to healthy controls. Moreover, the genetic material collected for this metagenomic analysis derives from prior isolation of virus/phage particles, arguing against the possibility of using latent DNA for viral identification and evaluation of abundance variation.

Our metagenomic analysis identified a diverse virome within the murine gut, comprising viruses that infect bacteria (bacteriophages), archaea (archaeal viruses, or archaeophages) and eukaryotes (eukaryotic viruses). As established in prior studies [67], bacteriophages constituted most of the viral community. However, we also detected a significant proportion of eukaryotic viruses, with a notable presence of giant viruses from the families *Mimiviridae*, *Phycodnaviridae*, *Marseilleviridae*, and *Iridoviridae*.

The detection of giant viruses in the mammalian gut is consistent with a growing body of literature, as their sequences are routinely found in metagenomes from diverse eukaryotes, including vertebrates such as mice and humans [78]. In some cases, associations between these viruses and disease development have been suggested, as in the cases of the *Mimiviridae* (which have been linked to the development of pneumonia) and the *Phycodnaviridae*, e.g., chlorovirus ACTV-1, which has been associated with altered cognitive performance in mice and humans [79]. However, the exact mechanisms underlying such connections are still a matter of debate, as the canonical hosts for most giant viruses found in mammals are believed to be resident protozoa or algae cells [67]. Nonetheless, their presence in the gut can exert a significant biological effect, even if they cannot directly infect mouse cells. In fact, giant viruses can be sensed by the mammalian immune system, triggering potent inflammatory responses, as their complex virions are rich in pathogen-associated molecular patterns (PAMPs). For instance, mimivirus particles can be recognized by Toll-like receptors (TLR2 and TLR9) and nucleotide-binding oligomerization domain (NOD)-like receptors in immune cells, leading to the production of pro-inflammatory cytokines such as TNF-α and type I interferons. This direct immune activation can occur through endocytosed viral particles or exposure to viral components from lysed protists, providing a direct link between the presence of giant viruses and the systemic inflammation that drives cachexia. Furthermore, some giant viruses can directly influence host physiology, as in the case of the *Iridoviridae*, which have been suggested to be a risk factor for type-1 diabetes by producing viral insulin-like peptides (VILPs) that bind and activate human insulin receptors [80, 81]. Thus, giant viruses have been shown to play important roles in sustaining a state of chronic inflammation and modulating host metabolism, both hallmarks of cachexia. We therefore speculate that the expansion of giant viruses observed in our CC animals may represent a previously underappreciated axis of pathogenesis, actively contributing to the inflammatory and metabolic disruptions that characterize the onset of cachexia (Fig. 6A).

The most pronounced shifts detected in the bacteriophage community of CC animals were significant reductions in the abundance of members of the families *Microviridae* and *Inoviridae*, which are core constituents of a “healthy” gut virome [82, 83]. This finding is consistent with a conserved pattern of virome depletion in gut inflammation, as decreases in members of the family *Microviridae* have been specifically documented in Crohn's disease, ulcerative colitis [84, 85], and *Clostridioides difficile* infection (CDI) [86]. Similarly, a reduction in *Inoviridae* members has been observed in the gut virome of Malawian children with severe acute malnutrition [87], a condition often associated with enteric inflammation and dysbiosis. Thus, depletion of these phages may disrupt the "top-down" predatory pressure exerted by the virome, which helps maintain bacterial diversity and community stability in “healthy” gut microbiomes [88, 89]. Thus, reduced populations of *Microviridae* and *Inoviridae* may contribute to the unchecked proliferation of pathobionts or specific bacterial consortia that contribute to inflammation and metabolic dysfunction (Fig. 6B), thereby exacerbating the cachectic drive. In this model, the loss of these phage taxa represents a failure of key regulatory mechanisms, facilitating the bacterial dysbiosis that fuels systemic inflammation [90].

Additionally, systemic inflammation – a key factor in CC development can occur via bacteriophage-mediated bacterial lysis [91]. The expansion of virulent phages (such as *Herelleviridae*) can lead to widespread lysis of their bacterial hosts. This process releases a flood of microbe-associated molecular patterns (MAMPs), including lipopolysaccharide (LPS) and bacterial DNA, which translocate across a compromised intestinal barrier. These MAMPs activate host pattern-recognition receptors (PRRs), triggering a cascade of pro-inflammatory cytokines such as TNF-α and IL-6, which are well-established mediators of muscle catabolism and adipose tissue wasting [92]. Moreover, it has been demonstrated that phage alone can induce severe colitis, as viral particles can directly activate TLR9 on dendritic cells after translocation to subepithelial tissues, leading to IFN-γ production and propagating inflammation systemically [93]. This inflammatory environment can in turn fuel further virome dysbiosis. Most gut phages originate as prophages integrated into bacterial genomes [88], and inflammatory mediators such as reactive oxygen/nitrogen species can induce the bacterial SOS response, reactivating prophages into the lytic cycle and increasing free phage populations by up to 10^5^-fold [94]. This creates a pernicious feedback loop in which inflammation drives phage expansion, which in turn exacerbates inflammation through bacterial lysis and direct immune activation, as shown in Fig. 6C (adapted from [93, 95–98]).

In this scenario, it is worth discussing the potential expansion of *Herelleviridae* in the gut of CC animals. While the low statistical power for this finding (~ 0.65) necessitates cautious interpretation, *Herelleviridae* are virulent phages that can contribute to this inflammation feedback loop. Moreover, they have a narrow host range, typically targeting *Firmicutes* bacteria [75]. The role of *Firmicutes* in the development of CC is particularly interesting, since studies conducted with different murine models have reported both reduction and expansion of this bacterial population in CC animals [8, 9]. We propose that these contradictory findings may be reconciled by considering the temporal dynamics of the *Herelleviridae-Firmicutes* relationship, which is likely governed by cyclical "boom and bust" population dynamics [99]. In this model, an initial proliferation (a "boom") of specific, susceptible *Firmicutes* populations could trigger the concomitant expansion of *Herelleviridae* through the induction of resident prophages integrated into the bacterial genomes. This early phase of viral replication would coincide with, or immediately follow, a period of high *Firmicutes* abundance in the gut of CC animals. However, the subsequent predatory activity of these now-numerous virulent phages would inevitably lead to the widespread lysis of their *Firmicutes* hosts, causing a population "bust" and resulting in the net reduction of this phylum, presumably observed in later-stage disease. This oscillatory predator-prey dynamic is a fundamental ecological principle and has been directly documented in microbiome studies, which have shown clear cyclical fluctuations between bacteriophages and their bacterial hosts [89, 100–102]. Therefore, the reported *Firmicutes* abundance is likely highly dependent on the timeframe of analysis, with different studies capturing snapshots of either the "boom," the "bust," or the transition between them. It is crucial to recognize that the *Herelleviridae-Firmicutes* dynamic is likely to be one of several concurrent phage-bacteria interactions within the dysbiotic gut. This complexity underscores the necessity of evaluating the gut virome and microbiome across multiple time points during cachexia progression, as a single snapshot may capture only a fixed frame of a highly dynamic ecological conflict.

A significant methodological limitation encountered in this study was the exclusive detection of DNA viruses, despite employing a protocol theoretically capable of identifying RNA viruses as well [26]. This absence is a common challenge in virome studies and can result from the lower abundance and higher lability of RNA virus particles, inefficiencies in the RNA extraction or reverse transcription steps, and the overwhelming background of ribosomal RNA from both host and microbiota components, which can obscure viral signals [82]. The failure to characterize the RNA component of the virome presents a critical caveat, as it results in an incomplete and potentially biased ecological picture. Thus, viruses that are important for gut health and systemic immunity, such as enteroviruses, noroviruses, or astroviruses, which are also known to disrupt barrier functions and trigger inflammatory pathways, remain undetected [13, 103]. Consequently, our findings are likely to represent only a partial view of the viral dysbiosis associated with cachexia, and the potential contributory role of RNA viruses in the observed pathophysiology cannot be ruled out. Future studies incorporating targeted RNA-enrichment strategies, such as probe-based depletion of ribosomal RNA or the use of specialized viral metatranscriptomic protocols, are essential to elucidate the role of the entire enteric virome in this wasting syndrome.

In conclusion, our study provides a preliminary characterization of the DNA gut virome in a murine model of Lewis lung carcinoma–induced cachexia, revealing a distinct dysbiotic signature marked by the expansion of eukaryotic giant viruses (mainly *Phycodnaviridae*) and depletion of core bacteriophage families, such as *Microviridae* and *Inoviridae*. These signatures bear relevant similarities to virome alterations reported previously in diseases also associated with persistent gut inflammation, such as ulcerative colitis, Crohn´s disease, and CDI [14, 84–86, 104, 105].

These findings demonstrate that viral communities are dynamically associated with the cachectic state, suggesting that they are an integral component of disruption of the gut ecosystem. We need to emphasize, however, that the preliminary nature of our study only establishes correlation. To move beyond correlation, direct experimental manipulation of the virome (through methods such as fecal virome transplantation or phage cocktail administration) will be critical to establish a causal link. Furthermore, the generation of robust cross-study viromic signatures will require a concerted effort across the field. Future studies should include integrated meta-analyses that aggregate data from multiple cohorts, similar to large-scale initiatives that have successfully defined core bacterial dysbioses in conditions like inflammatory bowel disease and colorectal cancer [106, 107]. Such meta-analytic frameworks provide a powerful model for determining conserved, cross-model virome alterations in cachexia, distinguishing them from model-specific or study-specific artifacts.

To this end, alternative models of cancer cachexia must be tested, including the use of female subjects to evaluate sex-specific virome alterations. Finally, while our findings provide a relevant basis to understand the roles played by the virome in CC development, significant caveats exist regarding their direct translation to human disease. The human gut virome develops under distinct dietary, genetic, and environmental pressures, resulting in a community that is qualitatively different from that of laboratory mice [82, 108]. Moreover, the high degree of inter-individual variation in humans means that identifying a consistent cachexia-associated virome signature will be a considerable challenge [82]. Thus, future clinical studies are essential to determine if phylogenetically or functionally analogous viral shifts are present and functionally relevant in patients suffering from cachexia.

## Electronic Supplementary Material

Below is the link to the electronic supplementary material

Supplementary Material 1

Supplementary Material 2

Supplementary Material 3

Supplementary Material 4

Supplementary Material 5

Supplementary Material 6

Supplementary Material 7

Supplementary Material 8

## Data Availability

All data (including images, scripts, raw sequences, and processed vOTU tables generated during the current study) were deposited at Open Science Framework - OSF (https://osf.io/at3xz). The captions for the Electronic Supplementary Material are provided in ESM_8.
