# Supplementary figures and images for: Dysbiosis of the enteric DNA virome correlates with the development of cachexia in a murine Lewis lung carcinoma (LLC) model
Source: Arch Virol. 2026 Feb 22;171(3):90. doi: 10.1007/s00705-026-06522-7 (PMC12926250; doi:10.1007/s00705-026-06522-7)

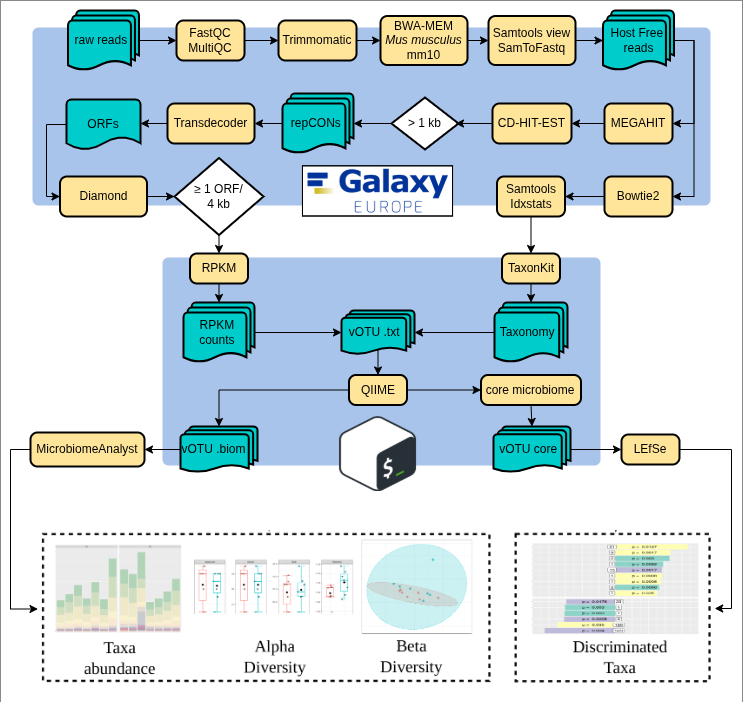

Supplement: Supplementary file 1 — Supplementary Material 1 [file 705_2026_6522_MOESM1_ESM.png]

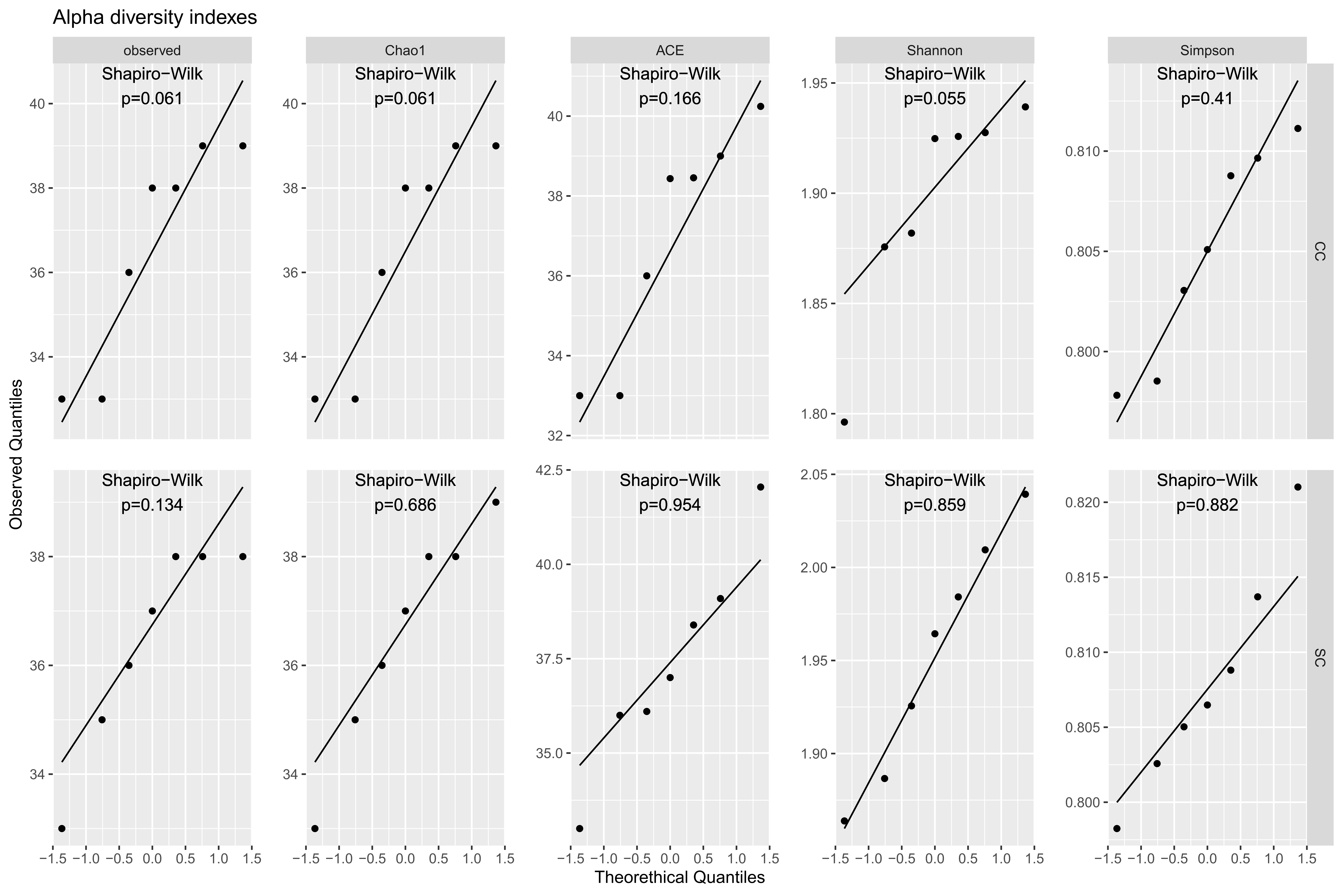

Supplement: Supplementary file 4 — Supplementary Material 4 [file 705_2026_6522_MOESM4_ESM.png]
