# Supplementary material for: Dysbiosis of the enteric DNA virome correlates with the development of cachexia in a murine Lewis lung carcinoma (LLC) model
Source: Arch Virol. 2026 Feb 22;171(3):90. doi: 10.1007/s00705-026-06522-7 (PMC12926250; doi:10.1007/s00705-026-06522-7)
Supplement: Supplementary file 8 — Supplementary Material 8 [file 705_2026_6522_MOESM8_ESM.docx]

Article title: Dysbiosis of the Enteric DNA Virome Correlates with the Development of Cachexia in a Murine Lewis Lung Carcinoma (LLC) Model

Journal: Archives of Virology

Authors: David Aciole Barbosa**^1*#^**, Yara N.L.F. de Maria**^2^**^*^, Fabiano B. Menegidio**^3^**, Regina Costa de Oliveira**^1^**, Daniela L. Jabes**^2^**; Luiz R. Nunes**^4^**

ORCID - DAB:0000-0003-3875-2307; YNLFM: 0000-0001-5249-1882; FBM: 0000-0002-4705-8352; RCO: 0000-0002-2446-5510; DLJ: 0000-0001-7297-0784; LRN: 0000-0001-9619-269X

**^*^**These authors have contributed equally to this work and share first authorship

Author affiliations:

**^1^**EasyOmics Biotechnology, Mogi das Cruzes, Brazil

**^2^**Núcleo Integrado de Biotecnologia, Universidade de Mogi das Cruzes, Mogi das Cruzes, Brazil.

**^3^**Núcleo de Pesquisas Tecnológicas, Universidade de Mogi das Cruzes, Mogi das Cruzes, Brazil.

**^4^**Centro de Ciências Naturais e Humanas, Universidade Federal do ABC, Santo André, Brazil.

**Electronic Supplemmentary Material (ESM) captions:**

ESM_1-workflow.png: Workflow applied to explore virome in cancer-cachexia.

ESM_2-readcounts.txt: Sequencing metrics of each sequenced sample.

ESM_3-R-script.R: R script containing code applied to data analyses.

ESM_4-normality.png: Normality test and qq-plots of alpha-diversity measures.

ESM_5-sample-data.txt: Sample data to input at MicrobiomeAnalyst.

ESM_6-otutable.biom: OTU-table-like in biom format.

ESM_7-otutable-core70.biom: OTU-table-like in biom format after core 70% filter applied.

ESM_8-Supplemental_material_captions.docx: Captions for ESM
